# Today and Future of Age-Related Macular Degeneration

**DOI:** 10.5402/2012/480212

**Published:** 2012-04-04

**Authors:** Kang Liu, Bolin Xie

**Affiliations:** Department of Ophthalmology, Kunming General Hospital of PLA, Yunnan, Kunming 650038, China

## Abstract

Age-related macular degeneration (AMD) is the leading cause of blindness in people over 50 in developed countries. Understanding of the pathologic process, genetic mechanisms, and risk factors of this disease has the benefit of seeking newer and more effective treatment options. Current clinical therapy for AMD shows a dramatic change from a decade ago. Anti-VEGF drug therapy is regarded as the more effective treatment for neovascular AMD now, especially combining PDT therapy. In the future, the genetic and biochemical therapies may be the promising treatments for AMD. This paper will focus on the progress of pathology, candidate genes of AMD, risk factors, and the existing drugs or surgical therapies available, in order to present some new directions of care with the prospect of improved vision in many patients suffered from AMD.

## 1. Introduction

Age-related macular degeneration (AMD) remains one of the most severe and profound disabilities encountered in medicine, particularly due to the loss of the central vision and the high economic burden it places on patients and societies. AMD is regarded as the most primary cause of blindness, particularly irreversible blindness [1]. The world Health Organization (WHO) summarised the most recent reports which the visual impairment was caused by many common ocular diseases, it was not difficult to find that AMD is the most common cause of blindness. AMD has resulted in the physical and mental health problems of a number of the geriatric population and their families tremendously. Recent years, it was not only high morbidity of AMD in western nations but also progressive incidence in eastern Asia areas, thus, continued research will be critically needed.

There were about over one hundred years for scientists to study AMD. Hutchison and Tay published an article named “symmetrical central choroidoretinal disease occurring in senile persons” in 1875. This may be the earliest medical literature about AMD that we can find. We reviewed the basic research towards AMD during about decade. There were three aspects should be summarized, including morphology, molecular biology, and genomics. AMD resulted from many dangerous factors, including genes and environment. With advancing age, some changes occurred in the retina, including alterations in retinal pigment epithelium (RPE) cellular size and shape, thickening of Bruch's membrane, thickening of the internal limiting membrane, and a decrease in retinal neuronal elements [2]. From a histopathologic standpoint, the initial morphological changes impacted on the photoreceptors and RPE cells, Bruch's membrane, and choriocapillaris in this multifactorial disease. The changes in the RPE and chioroid with age might provide a background for the development of AMD. An important pathological change in AMD was choroidal neovascularization (CNV). It was the defining feature of neovascular AMD, wet AMD, or exudative AMD. These new vessels growed into the subretinal space and caused classical pathological process of wet AMD [3]. Recently, the etiology of AMD kept a great many of unknown, numerous studies have suggested that genes are one of more important risk factors significantly impacted on AMD developing.

Ophthalmic genetics researches have carried out for about a decade, scientists tried to find the relationship between identification of risk factors associated with susceptibility to AMD and genes. A significant breakthrough in search of genetic contributors in AMD came in the spring of 2005. DNA sequence variant in complement factor H associated with AMD was found by four independent groups. They suggested that this variant, a nonsynonymous or disease relevant single nucleotide polymorphism (SNP), corresponds to a tyrosine to histidine polymorphism [4, 5]. Subsequently, LRP6 and VEGF were found that they may play a role in the risk of developing AMD [6]. But identification of the underlying genes was be difficult, with both genomic screen (locational) and candidate gene (functional) approaches being used.

Currently, not only in the developed countries but also developing countries, there are about 6.5% of people over 50 years who is affected by AMD. The underlying reasons for AMD rest with race, gene, age, gender, diet, smoking, education, cardiovascular disease, and sunlight group, and so on. In order to stop the progress of this disease, molecular mechanisms underlying AMD pathogenesis should be further focused on, and the need for novel therapeutic and preventative strategies for AMD is pressing. Established therapies for neovascular AMD, like photodynamic therapy (PDT) and anti-VEGF therapies, allow stabilization or even improvement of vision. Potential future drugs are under development for advanced AMD or its prevention targeted the signal transduction cascade of different angiogenic molecules. These drugs intervene at different levels of the involved processes including the RNA production and specific protein expression as well as inflammatory, apoptotic, or metabolic processes.

In this paper, we review the histopathologic findings that define AMD, along with new molecular pathologic findings that have advanced our understanding of the molecular mechanisms of AMD pathogenesis. Meanwhile, we aim at the current new therapies and to highlight possible future therapeutic strategies.

## 2. Pathology of AMD

The progression of AMD occurs over a few decades. Over the age of 70, the incidence of the disease increases dramatically. AMD is a heterogeneous group of disorders and exhibites the variability of symptoms, clinical findings, and natural history observed in patients. At present, it was known that AMD affects the neural retina, photoreceptors, RPE, Bruch's membrane, and the choriocapillaris. Clinically, early AMD is characterised by large drusen, go by the name of dry AMD. With the development of this disease, RPE pigmentary changes can progress to geographic atrophy. When the CNV was found in macular region, we defined the late stage as neovascular AMD or wet AMD. Although the pathogenesis of AMD remains greatly unclear, a complex interaction of genetic and environmental factors is thought to exist.

### 2.1. Early Stage AMD

Pathological changes of early AMD include the Bruch's membrane thickening and abnormal architecture, lipofuscin accumulation in the RPE, and drusen formation beneath the RPE in Bruch's membrane.

The earliest pathological changes are characterized by basal laminar deposits (BlamD) and basal linear deposits (BlinL). BlamD of AMD was located between the basement membrane of the RPE and its plasma membrane consists of basement membrane proteins and long-spacing collagen [7–9]. Subsequent reports confirmed that BlamD was the most prevalent histopathologic finding in early AMD eyes used as a marker for AMD grading [10], its thickness correlating well with the degree of RPE degeneration, photoreceptor fallout, and vision loss [11, 12]. The early BLamD clinically displays a normal fundus, and the late BLamD correlates most closely with clinical pigment abnormalities. It is the quantity and sites of membranous debris accumulation that appear to determine whether the disease develops pigment changes only or follows the alternative pathway of soft drusen formation with its attendant greater risk of CNV [13].

BlinL is made of vesicular material. It located in the inner collagenous region of Bruch's membrane. BlinL and BlamD are important positive associations with CNV, disciform scarring, and visual loss [11].

In early AMD, drusen are most frequently found as clusters within the macular region. If drusen diameter exceeds 25 mm, it may be visible ophthalmoscopically as yellow-white deposits [5]. Many laboratories studying these dumbbell shaped deposits have shown that drusen was composed of glycoproteins and lipids and accumulates in extracellular subretina often be located between the RPE basal lamina and Bruch's membrane. Clinically, drusen is classified morphologically either as hard or soft. Hard drusen exhibits some small, yellowish, and punctuated deposits. But it is not considered a particularly important risk factor for the development of AMD, especially drusen that is few in number and hard in quality. Hard drusen exists in common elderly patients without AMD also. In contrast, soft drusen presents a more diffuse, paler, and larger appearance, perhaps with “fuzzy” or blurry edges. The large, bilateral, and/or numerous soft drusen is significant risk factors for development of late stage AMD. Dynamic remodelling processes of drusen resorption and new drusen formation are maybe a useful marker of disease activity [14].

Although AMD is not a classic inflammatory disease, inflammation has been found to have an important role in disease pathogenesis and progression. Hageman et al. suggest that immune system may be involved in AMD pathogenesis through studies about inflammation-related molecules in drusen [15–17]. A recent research pointed out that recruitment of leukocytes and activation of the complement cascade occurred in mouse RPE and choroid during the process of age-related changes [18]. Hence, some researches targeted some specific inflammatory molecules, complement components, lipids, lipoproteins B and E, and glycoproteins, which are common constituents of drusen, in an attempt to better understand and treat AMD. Therefore, some reports deemed that anti-inflammatory agents such as corticosteroids, nonsteroidal anti-inflammatory drugs (NSAIDs), and immunosuppressive agents mighty provide an adjunct or alternative mechanism to suppress the inflammatory processes driving AMD progression [19].

### 2.2. Late Stage AMD

Late stage dry AMD is clinically characterized by geographic atrophy, which shows roughly oval areas of hypopigmentation and is often the consequence of RPE cell loss. Death of RPE cells led to the gradual degeneration of nearby photoreceptors. Subsequently, the retina became thin, going with progressive vision impairment. At the periphery of these hypopigmented areas, hyperpigmentary changes associated with compensatory RPE cell proliferation [20].

Late stage wet AMD attributes to choroidal neovascularization. Thickening of RPE basal lamina, decreasing of number of RPE cells and fundus structure impairment indicate the growth of choroidal new blood vessels [9–11]. Subretinal neovascular fibrous tissue can be found at macular area. The neovascularization has two different patterns: firstly new capillary vessels sprout from the choroidal vessels and penetrate through barrier formed by Bruch's membrane. Secondly, new vessels derive mainly from the retinal circulation, and they extend outward into the subretinal space. These vessels develop sometimes anastomose with the choroid-derived vessels [21–23]. It is called retinal angiomatous proliferation (RAP). However, RAP is much less frequently than CNV.

## 3. Candidate Gene of AMD

The AMD is greatly influenced by genetic factors in the pathogenesis, and genetic mechanisms underlying this disease are complex. Studies with environmental, genetic, and systemic health differences in development of AMD would provide a new understanding of the dynamic interplay between genes and environment. Researches have indicated a numer of AMD-associated genes in mechanistic pathways related to DNA repair, complement system activation, microglial recruitment, inflammation, neovascularization, and extracellular matrix function [24].

In 2005, independent research groups across the United States published a breakthrough identifying a DNA sequence variant in complement factor H that is associated with AMD. They found that single nucleotide polymorphism corresponding to a tyrosine to histidine polymorphism at position 402 which profoundly raise the odds for developing AMD [25, 26]. great many previous studies about genes have focused on SNP. Apparently, SNP and AMD have highly significant statistical associations, but they cannot account for the entire genetic component of the disease. Besides, there are some questions which whether the identified SNP is causally related to deviant gene function or whether it is plainly genetic markers. Conrad et al. reported another type of genetic structural variation called copy number variation, which has been known as an authentic source of phenotypic variation in human populations and be suitable for quantitative characterization [27]. But these methods did not find strong evidence related with AMD-associated genes.

So far, we found the main candidate genes related with AMD as follows: fibulin 5, CST3, CX3CR, TLR4, VEGF, LRP6, MMPs, HLA family of genes, and CFH.

Fibulin 5 is a 52-kDa calcium-binding epidermal growth factor (cbEGF-) rich extracellular matrix protein that is essential for the formation of elastic tissues. Ten fibulin 5 sequence variants have been associated with AMD and two other fibulin 5 mutations caused autosomal-recessive cutis laxa. Fibulin-5 was localized to BlamD beneath the RPE as well as some small drusen [28–30].

Cystatin C is a cysteine protease inhibitor that regulates the activity of cathepsin S, a protease with central regulatory functions in retinal pigment epithelial cells. Genotyping data suggest an increased susceptibility for AMD in CST3 B/B homozygotes. CST3 B may be a recessive risk allele [31].

CX3CR1 is expressed on retinal microglia cells and plays an important role in the activation and migration of the microglia [32]. Some reports deem that CX3CR1 is associated with wet AMD in Han Chinese and Caucasian population [33].

TLR4 (toll-like receptor 4) genetic variant alters the extracellular domain of the receptor, which interrupts the signaling transduction cascade and interferes with the expression of genes such as TNF-a, IL-1, IL-6, monocyte chemoattractant protein-1 (MCP-1 or CCL2), and its cognate receptor C–C chemokine receptor-2 (CCR2) [34]. Some researches suggest TLR4 as a candidate gene for AMD [35].

VEGF (vascular endothelial growth factor) is a major player in the control of angiogenesis and has a strong role to play in neovascularization. Increased expression of VEGF gene in the RPE and in the outer nuclear layer is associated with wet AMD [36, 37].

LRP6 (low-density lipoprotein receptor-related protein 6) is involved in vasculature remodeling pathways. Some data show LRP6 may play a role in the risk of developing AMD [6].

MMP (matrix metalloproteinase) is zinc-dependent endopeptidases; other family members are adamalysins, serralysins, and astacins. The MMPs belong to a larger family of proteases known as the metzincin superfamily. MMPs are also thought to play a major role on cell behaviors such as cell proliferation, migration (adhesion/dispersion), differentiation, angiogenesis, apoptosis, and host defense. Reduction in the levels of activated MMPs −2 and −9 may be responsible for impaired matrix degradation of Bruch's membrane leading to the pathology associated with AMD [38].

HLA (human leukocyte antigen) complex helps the immune system distinguish the body's own proteins from proteins made by foreign invaders. The HLA-Cw0701 allele, KIR haplotype AA, and HLA-B27 are associated with AMD [28, 29, 39].

CFH (Complement Factor H) mediates a number of essential biological functions that participate in host defense against infection, initiation of the inflammatory reaction, processing, and clearance of immune complexes, and regulation of the immune response. CFH rs3753394, rs800292, rs1410996, rs800292, rs1061170, and rs1329428 that are the AMD-associated single-nucleotide polymorphisms showed a significant association with wet AMD [33, 40–42].

## 4. Current AMD Clinical Researches, Therapies, and Prevention

### 4.1. Epidemiology

Many population-based epidemiologic studies examined age-related macular degeneration (Table 1) [43–49]. Recently, a USA nationally representative, population-based, and cross-sectional study involving a total of 5553 persons reported that the estimated prevalence of any AMD and late AMD, was respectively, 6.5%, 0.8% in USA population aged 40 years and older. But there is a significantly lower prevalence of any AMD in non-Hispanic black persons aged 60 years and older [50]. Another population-based cross-sectional study among elderly residents in Northeast Italy, 1162 randomly selected subjects aged 61 years or more were estimated to affect 62.7% of the whole population. It is not difficult to demonstrate that early AMD often be found over 50 ages. With advancing age, the prevalence of late AMD gradually increases. There are different prevalences in different area of the world.

In our views, more researches should necessary understand the underlying reasons for the different patterns of presentation of early and late AMD among race, genes, age, gender, diet, smoking, education, cardiovascular disease, and sunlight groups in order to explore the predictive value of early AMD to stop the progress of this disease in different populations.

### 4.2. Risk Factors

Great many risk factors have been reported to associate with AMD, such as age, iris color, cataract/cataract surgery, cardiovascular factor, hypertension, diabetes, arthritis, diet, fat, drug, gene, smoking, and family history of AMD. But the evidence and strength of association are variable. Recently, a systematic review, prospective and cross-sectional studies, involving 113,780 persons with 17,236 cases of late AMD, estimated the association between late AMD and risk factors [51]. These results of meta-analyses show that risk factors of late AMD with strong and consistent associations were increasing age, current cigarette smoking, previous cataract surgery, and a family history of AMD; moderate associations were higher body mass index, history of cardiovascular disease, hypertension, and higher plasma fibrinogen; weaker associations were gender, ethnicity, diabetes, iris colour, history of cerebrovascular disease, serum total and HDL cholesterol, and triglyceride levels. 

### 4.3. Therapies

We review current therapeutic methods and discuss possible future strategies about AMD in this part. Dry AMD possibly translates into wet AMD at every phase in developing AMD. Especially, the neovascular exsudative form of AMD may result in severe sight loss. Although therapies for AMD were limited to some extent, new therapies have emerged for both the nonexudative and exudative AMD, which have improved prognostic outcomes. These treatments include nutritional supplementation, antioxidant prophylaxis, intravitreal injection of medications that inhibit aberrant vascular proliferation, PDT, and transpupillary thermotherapy (TTT). However, monotherapy for AMD, especially wet AMD, produces very little effect currently. Thus, choosing of the optimal therapeutic schedule is a burning question. Presently, combination of different therapies is the best therapeutic plan for doctor to select, but method of combined treatment and consolidation treatment of followup remains a problem to be solved for wet AMD. 

#### 4.3.1. Noninvasive Therapies

Antioxidant therapy, particularly in nonexudative age-related macular degeneration, may plays a role in minimizing disease progression. The Age-Related Eye Disease Study (AREDS), a multicenter, double-masked, randomized, controlled clinical trial revealed that oral supplementation of a combination of vitamin C, vitamin E, beta-carotene, zinc oxide, and cupric oxide in patients with advanced AMD in one eye had a 25% relative risk reduction of developing advanced AMD in the other eye. The relative risk of vision loss of three or more lines was reduced by 19% [34]. However, there was no evidence to support that vitamin supplementation is good for patients with early nonexudative AMD or active exudative AMD in both eyes. Surprisedly, the research of the Eye Disease Case-Control Study Group demonstrated that increased intake of vegetable fat, mono- and polyunsaturated fatty acids, and linoleic acid has also been associated with an increased risk for AMD [52]. Conversely, higher intaking of fish, nuts, and dark green leafy vegetables has been associated with a lower risk for AMD [35]. 

TTT is a brachytherapy which uses of a long-pulse, 810 nm near-infrared laser irradiation. TTT can produce approximately 10°C for typical clinical parameters used in CNV treatment. But, the treatment effectiveness of TTT is uncertain, and it has complications, with posttreatment haemorrhage and macular infarction having been reported [28, 39]. It is not generally used worldwide now. 

#### 4.3.2. Invasive Therapies

PDT is relatively effective invasive therapy for exudative AMD. The evolution of subfoveal choroidal neovascularization was more than that of juxtafoveal choroidal neovascularization after PDT treatment. Owing to the former, it grew quickly toward the fovea and visual acuity loss was greater. PDT is a safe, long-term treatment for exudative age-related macular degeneration [53], but it is not definitive because this treatment cannot stop the initial growth of the choroidal neovascularization lesion [54]. 

Intravitreal ranibizumab and bevacizumab monotherapy were regarded as the more effective treatment for neovascular AMD now, they go by the name of antivascular endothelial growth factor (anti-VEGF) therapy. 

Bevacizumab is a full-length monoclonal antibody that binds to and blocks the action of all VEGF isoforms. Numerous studies towards intravitreal bevacizumab have reported its efficacy for neovascular AMD and low rates of treatment related complications [55–57]. 

Ranibizumab is a humanized antigen-binding fragment targeted all isoforms of VEGF-A. At present, clinically, it had been approved for the treatment of all subfoveal CNV subtypes secondary to AMD [58, 59]. Researches pointed out that patients who initially received three injections of 0.5 mg ranibizumab intravitreously approximately 4–6 weeks apart may improve visual acuity [59]. 

One study towards intravitreal bevacizumab (Avastin) versus ranibizumab (Lucentis) demonstrated that the bevacizumab had too many methodological limitations to rule out any major safety concerns, and higher evidences from ranibizumab trials hinted signals for an increased ocular and systemic vascular and haemorrhagic risk which warrants further investigation [60]. 

The advent of anti-VEGF therapy was revolutionary in the treatment of wet AMD. But the pathophysiology of CNV involved both angiogenesis and vasculogenesis [29, 30]. However, the effect of anti-VEGF therapy was limited to the angiogenic component of CNV. Evidence suggested that the treatment response may diminish as maturation of the CNV occurs [31, 32]. In contrast, PDT therapy was possessed of the different mechanism to treatment CNV compared with anti-VEGF therapy, and it induced the occlusion of microvasculature within a CNV lesion. Therefore, in terms of prevalent views, main combining therapy incorporating PDT therapy and anti-VEGF therapy may be beneficial to yielding longer treatment-free intervals and requiring fewer intravitreal injections [53]. And the combination therapy may represent an alternative treatment approach and randomized multicenter clinical trials are ongoing [61]. 

#### 4.3.3. Surgical Intervene

Patients with wet AMD often suffer from large submacular hemorrhage, a major source of severe vision loss. With thickness of submacular hemorrhages, subretinal fibrosis, and widespread atrophy will occur in the retina. There are two options for the displacement of sub-macular hemorrhage. One is injection of an expansile gas in the vitreous cavity with or without simultaneous intravitreal injecting of tissue plasminogen activator (tPA), another is removing the vitreous gel and replacing it with expansile gas with or without intravitreal or subretinal injection of tPA. But these techniques applied to small patients [62, 63]. And the average final visual acuity of those patients in studies was poor, although the visual acuity improved transitorily after operation. Retrospective meta-analysis of removal of subretinal hemorrhage showed only modest improvement in visual acuity with a 25% recurrence rate [64]. The failure of surgical intervention attributed to largely macular atrophy after excision of CNV or evacuation of subretinal hemorrhage, which was unavoidable excise the RPE. 

#### 4.3.4. Possible New Treatments

At present, although current therapies for CNV may in some cases stop disease progression, but by and lage they do not successfully reverse visual loss in patients with AMD [65]. Significant progress has been made in the field of gene transfer to the eye, which now allows researchers to stably express nucleic acids in the different cell types that make up the eye. Such advances, which have been made primarily in the retinal degeneration field, may now be applied to provide a solution for the multitude of patients losing vision to ocular neovascularization. The mechanism of CNV is distinct and is not completely understood, they both involve an imbalance between pro- and antiangiogenic factors that leads to pathologic neovascularization [66]. The future of treating AMD appears promising particularly as we gain further insights into the genetic and biochemical pathways of the disease [66]. 

The development of a gene therapy regimen would offer not only the prospect of local delivery, but also local production of the antiangiogenic protein for a prolonged period of time. The strategy for treating any disease due to loss of function of a single gene is straightforward once the gene and the appropriate cell targets have been identified. There are now several different vectors available which have characteristics suitable for delivering large or small genes in a stable fashion to a variety of ocular cell types [67]. There are considerably more challenges in developing a treatment for diseases for which particular target stages/genes are ill defined. Thus, the primary challenge in developing gene therapy for CNV is not how to deliver the therapeutic gene but, rather, to identify the optimal molecule to deliver. 

Pigment epithelium-derived factor (PEDF) is a potent inhibitor of neovascularization [68]. Data have led researchers in the field to pursue the rescue of animal models of CNV with PEDF gene transfers to establish a therapeutic proof in principle [69, 70]. 

Encouraging results have been reported with the use of the adeno-associated virus (AAV) 2-sFLT01, a vector containing the vascular endothelial growth factor receptor, angiostatin, and most recently, pigment epithelium-derived factor (PEDF). AAV that expresses a modified soluble Flt1 receptor designed to neutralize the proangiogenic activities of VEGF is likely to treatment of AMD via an intravitreal injection, owing to well-toleration, localization, and capability of long-term expression of AAV2-sFLT01 [71]. VEGF soluble receptor gene transfection can inhibit subretinal neovascularization (SRN). This method will contribute to future gene therapy for AMD [72]. 

A soluble form of the VEGF receptor Fms-like tyrosine kinase (sFlt-1) has been shown to bind VEGF and blocks its signaling pathway [73]. 

Moreover, AMD is a highly multifactorial disease likely caused by individual-specific combinations of etiological and risk factors that affect not only the disease itself but also its response to various treatment modalities. Thus, future studies on the genetic and pathology of AMD might help doctors develop effective therapies and ultimately improve quality of life for AMD patient. 

## 5. Summary

AMD exhibited clinically loss of central vision and pathologically the accumulation of drusen, and RPE degeneration, photoreceptor atrophy and in some cases with CNV. Meta-analyses demonstrated that the current model of pathogenesis likely involves age, iris color, cataract/cataract surgery, cardiovascular factors, hypertension, diabetes, arthritis, diet, fat, drug, gene, smoking, and family history of AMD. The retina and RPE with progressive oxidative damage bring structural and biochemical changes that promote inflammation and angiogenesis. Although researchers know about some finite pathogenesis of AMD, the etiology and pathogenesis of the disease remain mainly unclear. Treatment options for different stage of AMD are similarly limited. So concomitant novel preventative and therapeutic strategies are necessary. 

Oxidative stress, inflammation, lipid metabolism, and angiogenesis play significant roles on the molecular pathology of AMD in recent researches. The retina and macula in particular are highly impacted on by the raised oxygen tension, high metabolic activity, optical radiation, and oxidizing processes of photoreceptor phagocytosis. Meta-analyses demonstrated that increased risks consistently associated with cigarette smoking, genetics, and a potent oxidizing stress and decreased risk associated with higher intake of fish, nuts, and dark green leafy vegetables. 

Although AMD is not a classic inflammatory disease, pathology of AMD shows signs of chronic inflammatory damage, including modest infiltration of some inflammatory cell and existence of inflammatory factors. The newest research findings prove a genetic link of the complement system. Compared to the scenario with complement, the definite role of some inflammatory cell, such as macrophages and microglia in AMD pathogenesis, is protective and harmful, it lacks of clear. 

Recent experiment of animal model and clinic of AMD towards VEGF suggested that a potential role of VEGF was a key angiogenic factor in the development of CNV in late stage AMD, and more researchers suggested that polymorphisms in the VEGF gene might bring about risk of AMD and effect on anti-VEGF therapy. Further investigations of the gene profiles of AMD patients may play important role to anticipate different therapeutic strategies. Potentially, genomics will guide new therapy regimes and fundamentally change the therapy and prognosis of AMD. From our view, along with our understanding accumulated from the field of molecular epidemiology, genomics, and pathology of AMD, future studies on the molecular pathology of AMD will help us gain further insights into the specific molecular signatures of AMD and ultimately find out an optimal way to cure patients of AMD. 

In our opinion, the future genetic tests should explain the correlation between the individual genetic risk and suffering from late AMD, in order to interfere in the progress of AMD early. Accordingly, more intensive/combination stratifies could be used with patients according to their special genotype. The gene therapy should be promising to treat all AMD. 

## Figures and Tables

**Table 1:** Epidemiological Studies of Age-related Macular Degeration (early AMD and exudative AMD): distribution by age groups (%), (percentages for exudative AMD are given in parentheses).

Study/age	50–59	60–69	70–79	Over 80
Beaver Dam (USA) 4756 patients	16.7 (0.5)	25.3 (1.4)	41.7 (6.9)	48.7 (13.5)
Proyecto VER (Hispanic people in Arizona) 2780 patients	19.8 (0.1)	27.7 (0.5)	41.3 (0.8)	54.1 (4.3)
Rotterdam (Netherlands) 6411 patients	2.5 (0.1)	9.9 (0.7)	16.7 (3.2)	29.8 (11.6)
Blue Mountains (Australia) 3585 patients	2.8 (0.2)	9.2 (0.7)	20.9 (5.4)	46.6 (18.5)
Reykjavik eye study (Iceland) 1021 patients	0.3 (0.0)	1.2 (0.0)	5.3 (0.5)	25 (9.8)
Beijing eye study (China) 4376 patients	1.4 (0.1)*			

*The Beijing Eye Study are not classified by age.

## Abbreviations

AMD: Age-related macular degeneration;
AAV: Adeno-associated virus;
BlamD: Basal laminar deposits;
BlinL: Basal linear deposits;
CFH: Complement factor H;
CNV: Choroidal neovascularization;
HLA: Human leukocyte antigen;
LRP6: Low-density lipoprotein receptor-related protein 6;
MMP: Matrix metalloproteinase;
PDT: Photodynamic therapy;
RAP: Retinal angiomatous proliferation;
RPE: Retinal pigment epithelium;
SNP: Single nucleotide polymorphism;
TLR4: Toll-like receptor 4;
tPA: Tissue plasminogen activator;
TTT: Transpupillary thermotherapy;
VEGF: Vascular endothelial growth factor.
